# Improving representation of Hispanic adults in a population-based cancer genetics cohort: Qualitative findings from the Healthy Oregon Project

**DOI:** 10.1017/cts.2024.544

**Published:** 2024-05-22

**Authors:** Gloria D. Coronado, Jennifer S. Rivelli, Vanessa Serrato, Jamie Thompson, Autumn Shafer, Danita Tracy-Carter, Kayla Warner, Jackilen Shannon

**Affiliations:** 1 Kaiser Permanente Center for Health Research, Portland, OR, USA; 2 Oregon Health & Science University, Portland, OR, USA; 3 School of Journalism and Communication, University of Oregon, Eugene, OR, USA

**Keywords:** Study recruitment, Latino/Hispanic/Latinx, genetics study, patient engagement

## Abstract

**Background:**

Members of many racial and ethnic population subgroups are underrepresented in clinical trials and research. We present perspectives on barriers and facilitators to study participation gathered from Hispanic participants in a population-based genetic screening study.

**Methods:**

Seven focus groups (five in English and two in Spanish) were conducted with self-identified Hispanic participants of the Healthy Oregon Project (HOP), a large population-based cohort of adults residing in Oregon. HOP study participants complete surveys about cancer and chronic disease risks with the option to donate a saliva sample for no-cost genetic risk screening for inherited disorders. HOP invited Hispanic participants via email to join a focus group about their experiences. Focus groups, generally lasting 60–90 minutes, occurred in person and virtually. Notes were coded and content-analyzed.

**Findings:**

49 Hispanic adults participated in the focus groups (37 women; 9 men; 3 non-binary people). Identified facilitators for HOP study participation were trust in the academic medical center leading the study, having a family member who was impacted by cancer, and receiving free genetic screening. Identified barriers were difficulty completing the family history survey, lack of understanding or familiarity with research, immigration status, and navigating technology challenges. Recommendations to improve recruitment of Hispanic populations included promoting the study at community events, clinics, or schools, simplifying the consenting process and providing patient-focused videos to demonstrate study tasks, providing real-time sample tracking, and offering monetary incentives.

**Discussion:**

Our findings can inform strategies for bolstering recruitment of Hispanic adults in biomedical research studies.

## Introduction

The underrepresentation of diverse participants in US clinical trials and research is a longstanding issue with significant societal and economic consequences. Recent reports reveal that non-Hispanic white individuals comprised 78% of participants in US clinical drug trials conducted between 2015 and 2019, despite making up less than 60% of the general population during this period [1]. A particularly stark example is the underrepresentation of Hispanic individuals, who constituted just 9% of participants in NIH-funded studies from 2012 to 2018, despite comprising 19% of the US population [2,3].

The consequences of this unequal representation are profound. It hinders innovation, recruitment success, and the generalizability of research findings. Moreover, it contributes to health disparities, erodes trust in medical research and the healthcare system, and results in significant economic costs [2]. The National Academies of Science, Engineering, and Medicine (NASEM) has called for a change, urging national organizations like the US Food and Drug Administration and the Department of Health and Human Services to enhance transparency and accountability in clinical trial and research study enrollment across demographic subgroups [2].

The barriers to participation among underrepresented subgroups are multifaceted, including individual and community characteristics, study-related factors, institutional policies, and the research landscape [2]. In a community-based survey on barriers and facilitators to research participation (among individuals who had not participated in a research study), Hispanic respondents commonly noted the need to care for family members (82%), lack of time (75%), fear of research-related costs (74%), low trust (71%), and the degree of hassle (73%) as barriers to participation [4]. Conversely, common facilitators were having a friend or family member impacted by the disease being studied (80%) and monetary compensation (73%) [4]. Apart from participant factors, research shows that study participation is influenced by investigator bias, community engagement, language proficiency of research staff, and user-friendly research processes [2].

Efforts have been made to increase diversity in clinical trials and research [5], but there’s a lack of research on successful approaches to recruit Hispanic participants, especially for studies involving genetic testing. Diverse participation in such research is critical for personalized treatments, for diseases such as cancer, ensuring they’re effectively tailored for a broad population [6].

The Healthy Oregon Project (HOP) is addressing this challenge as a statewide initiative led by the Oregon Health & Science University (OHSU) Knight Cancer Institutes’ CEDAR (Cancer Early Detection Advanced Research) Center. HOP aims to assemble a 100,000-person population-based cohort to understand how genes, environment, and behaviors impact health [7]. In this report, we explore the perspective of Hispanic HOP participants, shedding light on their motivations, barriers to participation, and suggestions for enhancing recruitment. These insights will be invaluable in guiding future efforts to diversify participants in clinical trials and research.

## Methods

### The Healthy Oregon Project

HOP is a statewide effort that aims to build a large research data repository containing survey and biological sample data on a population-based cohort and to provide personalized health information to participating cohort members by providing no-cost genetic screening for genetic variants associated with various cancers and heart disease (31 genes for inherited cancer and 1 gene for familial hypercholesterolemia) [7]. The overall goal of the project is to understand factors associated with cancer and other diseases by combining survey data on health, wellness, and behavior collected via smartphone application with genetic information from voluntarily collected biological specimens (e.g., saliva) through mailed kits. Data is saved to a secure and privacy-protected repository that can be used by researchers to answer many different questions about health. Genetic results are provided to participants at no charge: results are uploaded into the Health Insurance Portability and Accountability Act (HIPPA)-secure HOP app, while participants with a positive result are contacted directly by an OHSU genetic counselor to review results and be informed of medical guidelines.

HOP is led by OHSU scientists and collaborators across Oregon at multiple organizations, including the University of Oregon, Oregon State University, Providence Cancer Institute, OCHIN, the Oregon Health Authority, and Kaiser Permanente Center for Health Research. Study procedures were reviewed and approved by the Institutional Review Board of OHSU (IRB #18473). Pilot recruitment for HOP began in 2018 and primary recruitment began in October 2020. Recruitment is primarily conducted through paid advertising on social media, word of mouth, and community outreach events focused on health (not generally focused on Hispanic populations). No monetary incentives are offered.

To achieve its overarching goals, HOP must recruit a diverse population of participants, specifically to mirror the racial and ethnic composition of Oregon. So far, 40,000 people have joined the study, but the Hispanic enrollment remains lower than desired. Presently, only approximately 6% of current HOP participants identify as Hispanic, contrasting with the approximately 14% representation of Hispanics among the overall Oregon population [8].

For this study, we organized seven focus groups comprising HOP participants from rural and urban regions who self-identified as Hispanic or Latino. The objective was to gain insights into three key areas: (1) the motivations behind their decision to participate in HOP, (2) the challenges they encountered and the factors that facilitated their participation in the study, and (3) their feedback on how to enhance HOP’s recruitment strategies and messaging to attract Hispanic participants. The findings provide valuable strategies to enhance the recruitment of both Spanish- and English-speaking Hispanic patients, which will be directly implemented in the HOP study and can have broader applications in future research, especially in studies involving genetic screening components.

### Recruitment and data collection

We used a positive deviance approach to understand motivations, challenges, and facilitators and desired enhancements among participants in the HOP study [9]. The positive deviance approach to research subject recruitment provides a valuable method for identifying innovative solutions by learning from individuals who have successfully navigated similar obstacles, ultimately fostering community empowerment and sustainable change. The study team selected geographic regions, representing rural and urban/suburban locations that had relatively high Hispanic enrollment. Using the database of all HOP participants, study staff identified individuals who self-identified as Hispanic or Latino within the selected regions (based on zip codes) and sent them email invitations to participate in region-specific focus groups. The email contained a link to a study intake form to confirm their intent to participate and county of residence. Participants were offered the option to sign up for a group held in English or Spanish and could choose a location to attend in person or to attend a virtual focus group. They were offered a $100 gift card for participating in the focus group. Two groups were Spanish language only (one virtual, one in person at the Kaiser Permanente Center for Health Research), and five groups were offered in English (two virtual, three in person at the Knight Cancer Research Building and OHSU Primary Care Clinic). All focus groups were conducted in April of 2023.

The research team developed a semi-structured interview guide based on prior literature and input from the research team (Supplementary Table 1). Focus group facilitators followed the detailed guide and asked follow-up probes to explore each section in depth. Sections included questions about how participants heard about HOP and motivations for participating, facilitators and barriers to participation, the quality of the information they received about the HOP study, participants’ emotional responses to receiving and completing the saliva testing kit, what aspects of the research process they found respectful, overall trust in research and messengers, the influence of family in their decision to participate in HOP, and overall experience with the study and suggestions for improvement. Focus groups were conducted by trained and experienced qualitative staff (JSR, DTC, KW). Each focus group session lasted approximately 90 minutes, and depending on group size, one or two support staff were present to greet participants, take notes, and hand out gift cards. The facilitator conducted a verbal consent process. All interview materials and processes were reviewed and approved by the Institutional Review Board at OHSU.

### Analysis

All focus groups were audio-recorded and summarized in real time by a notetaker. The lead facilitator (JSR) created qualitative summaries of each focus group. Primary patterns in the data were noted and classified into key content themes, with particular emphasis on similarities and differences between language groups. Within each major theme, subthemes emerged, offering deeper insights into specific aspects of the data. Following each focus group, the session lead and co-facilitators participated in a review process, ensuring the accuracy and comprehensiveness of the data captured in the summaries. The review process served as a quality check, helping to mitigate potential biases and oversights, and an opportunity to assess whether thematic saturation had been reached. Summary themes and illustrative quotes were then reviewed by the larger research team for further input, resulting in finalized summaries.

## Results

Study staff identified and contacted 1,963 individuals via email. A total of 942 (48%) opened the email invitation, of whom 226 (24%) clicked the embedded link to learn more about the study and 81 (9%) signed up to participate. Ultimately, a total of 49 individuals participated in the seven focus groups (15 in rural regions, 34 in urban/suburban regions), and focus groups ranged in size from 4 to 11 individuals (Table 1). Discussions with the session facilitators revealed that thematic saturation had been reached after five focus groups were completed. Here we report a summary of the findings, noting differences in responses for English-language (EL) and Spanish-language (SL) speakers, where they were present; we report more detail on these differences in Supplementary Table 2. We do not report differences by urban-rural status, as we did not link responses to residency status in our mixed urban-rural focus groups.

**Table 1.** Description of participants in the seven focus groups (F1–F7)

English language groups	F1 (*N* = 7): 1 male, 5 female, 1 non-binary, 29–49 age range, 7 urban F2 (*N* = 9): 4 male, 5 female, 30–66 age range, 9 urban F3 (*N* = 11): 2 male, 9 female, age range 26–67, 4 urban/7 rural F4 (*N* = 6): 1 male, 4 female, 1 non-binary, 27–65 age range, 5 urban/1 rural F5 (*N* = 6): 0 male, 6 female, age range 20–68, 2 urban/4 rural
Spanish language groups	F6 (*N* = 6): 1 male, 4 female, 1 non-binary, 30–48 age range, 5 urban/1 rural F7 (*N* = 4): 0 male, 4 female, 22–52 age range, 2 urban/2 rural

### Motivators of study participation

Most participants said that they were motivated by having had personal experiences with either having cancer or having loved ones with cancer. For these participants, the opportunity to receive genetic screening at no cost was particularly motivating (Table 2). One participant whose parent recently passed away from cancer stated that she decided to participate in the study “for my daughter,” believing that a high risk of cancer could be carried in her family (EL, female). Once participants had agreed to participate in HOP, some noted that the sign-up process was quick and easy (22%) and some were motivated to participate by seeing the word “free” (14%). Other participants noted that OHSU is known and trusted in the community, which facilitated their participation and reassured them that the social media promotion was not “fake” or a “scam.”

**Table 2.** Summary of main themes and sub-themes

General motivators for participation		
**Main theme**	**Sub-theme**	**Total**
Established relationship with Oregon Health & Science University (OHSU)	Trusted name/organization, cares about the community	18
	Existing relationship with OHSU, that is, employee, volunteer, patient	2
Research benefit	Experience with research studies	11
	Latino/a representation in research, need more people of color involved	1
Personal connection to cancer	Personal experience with cancer, friends/family with cancer	19
	Family member/friend died of cancer	9
Family/friends engagement	Doing it for their family and future generations	5
	Completing kit with friends	4
	Encouragement from children, partner	2
Prevention	Fearful of unknown cancer history, ideal way of learning more	12
	Early detection and treatment	11
Participation process	Kit collection box at work	2
	Kit was easy to complete	5
Financial benefit	Free of cost	10
**Barriers to participation**		
**Main theme**	**Sub-theme**	**Total**
Navigating technology	Lack of access to social media platforms, unfamiliar with downloading applications	5
Family history	Difficult to answer family history questions on the survey	7
Distrust in research	Lack of understanding, unfamiliarity, or nervousness about research	6
	Medical distrust	2
Health literacy	Difficulty understanding the genetic information and consent	3
	Difficult for non-English speakers	2
Mailing process	Difficult to find mail drop box	2
Privacy concerns	Concern about access or sharing participant data, that is, immigration status	6
Fear	Fear of results	4
	Fear of the unknown – myths about cancer, lack of education and awareness	1
Financial concerns	Unsure about the cost, no insurance	1
**Recruitment recommendations**		
**Main theme**	**Sub-theme**	**Total**
Print	Posters, brochures in trusted locations, that is, clinics, urgent care, Latino community organizations	12
	Mailed brochures to homes	3
	Posters on buses, trains	2
	Posters on college campuses, wellness centers	2
Digital	School district emails to families	10
	Email advertisement circulated at work	3
In person	Tabling at community events, health fairs, medical clinics, Women, Infants, and Children (WIC) programs, word of mouth, Latino grocery stores (include demonstration of kit)	26
	Religious organizations	4
	Promotoras de Salud/health educator event	3
Broadcasting	Radio, TV ads	2
Provider recommendation	Provider recommendation to participate in genetic testing	9
Eligibility	Open registration to non-Oregon residents	2
Benefits	Free of cost	7
	Incentives, that is, gift card, raffle, zoo pass	6
	Awareness, importance of participating	6
	Focus on children/family as motivators	5
**Study design recommendations**		
**Main theme**	**Sub-theme**	**Total**
Materials	Disclaimer about whether not knowing family history impacts results	6
	Simplify the consent form and results summary	6
	Reduce the length and redundancy of surveys	3
	Emphasize OHSU in materials	1
Healthy Oregon Project application	Offer alternative options for sign-up and survey completion (i.e., computer, tablet, in person, etc.)	7
	Simplify app steps, smoother transitions	5
Videos	How to complete the kit and how it gets processed	7
	Educational video about genetics by a provider	5
	User experience testimonial	2
	How to download the app and sign up	1
Genetic screening kit	Additional graphics, detailed instructions	2
	Options for individuals without housing	1
Follow-up support	Resources after an abnormal result	7
	Kit tracking, that is, email or text notifications	3
	Results summary should include guidelines/recommendations on other preventive screenings	2

### Barriers to study participation

Several barriers to participation were reported. Some participants thought the study consent form could overwhelm or deter participation in the study, especially for those having a low level of educational attainment. One participant noted, “The research consent [form] has a lot of information, and there are people who may not understand due to their education level. Some people could find it difficult. Maybe when they get to that point, they may decide against signing up. For example, my husband may not sign up if he had to read all that information.” (SL, female) Participants also noted some barriers to providing complete information on surveys after enrolling. Specifically, some participants experienced difficulty filling out family history questions, especially when they did not have complete information about their family history. Participants expressed concerns that not knowing their family history could diminish the value of genetic screening. One respondent noted, “I don’t know a lot of my medical family history so I was a little concerned that because of that my results would not be entirely reflective of my true genetics” (EL, female).

Lack of understanding or familiarity with research was mentioned as a barrier. One participant noted, “My parents are from Mexico. My father does not go to the doctor, and my mother does. My mother understands how to do research and use a computer, and my father does not. These differences can exist in the same generation” (SL, male). Concerns regarding immigration status were also mentioned by 12% of participants. One participant noted, “Some communities are afraid to sign up, afraid of what authorities could end up finding out. Despite saying your information is protected, what this means needs to be better defined so that participants can make an informed decision” (SL, female). Another barrier was navigating technology, such as downloading the app and having access to social media and email. One participant stated, “Why do we have to download an application? This could be a barrier. Downloading is another step. I did not have to think about it much, but some people may not be able to do this” (SL, female). Other barriers, such as having competing priorities or having language barriers, were also reported. As one participant noted, “Some people do not speak English or do not have insurance. They have jobs, kids, and are tired” (SL, female).

### Emotional responses to participation

Several respondents reported an emotional response to some aspect of participating in the study. Some participants, especially those who were cancer survivors, reported feeling anxious about signing up for the study and learning about their screening results. Although participants were informed that there would be a six-month waiting period between when they complete their sample and their results are returned at initial recruitment, sign-up, and on the HOP app and email newsletters, some participants stated that the wait led to anxiety and was described as “too long to wait.” One participant noted, “The waiting process made me a little anxious. When I got the results, notifications and emails were flat. A lot of “build-up” for not much information” (EL, male). Another participant stated, “…after you take the test, there’s a little concern until you get your results back. I was thinking about the next steps, how would I talk it over with my family, if something was wrong” (EL, female). The long wait to receive screening results led to worries that participants had mishandled the sample or mailing process, and some worried about completing the kit incorrectly or that the sample may have been damaged in the mail. Finally, some participants reported feeling confident and a sense of safety about participating in the study, reporting that “the more you know, the more confidence you have….”

Regarding feelings of respect (respeto), which is an important Latino value, participants generally reported that they felt respected by the study materials, which were designed to be accessible to a wide range of reading levels, and that the study’s customer service was timely and respectful. Some participants said that their participation in the study led them to feel that OHSU cares about the community.

Regarding feelings of trust (*confianza*), participants reported that they trusted getting genetic information from OHSU and their providers. Family (*familismo*) is another important value for many members of the Latino community. Eighteen percent of participants reported that having a family member or friend pass away from cancer had an influence on their decision to participate. Nearly one-quarter (24%) reported that they spoke with other family members about their experience and their results. When asked what advice they would share with their families, 24% said they would educate family members on the importance of early detection and taking early action to get screened, and others said that they inform their family members that the genetic screening offered through the study is free.

### Recommendations for improving recruitment

To improve recruitment of Hispanic individuals, focus group participants suggested promoting HOP at community events (attended by Hispanic adults), hanging posters and handing out flyers at community clinics and events, or sending home brochures with school children. One participant suggested, “There could be a tabling event, or sign-up available at stores” (SL, female). Participation in community events was thought of as “meeting people halfway.” Focus group participants felt that in-person promotion, sign-up, and consenting could address participants’ questions and concerns and make the process less overwhelming. One person recommended an event similar to a hospital-based event they had previously attended, “In a hospital in Oregon there is a group that was distributing boxes with food and COVID tests. That seems like a good idea to apply to HOP. Have pamphlet, instructions, test kits, inside. It would be good to have a person there to respond to questions that someone has.” (SL, female) Other participants noted that group-based promotional events could be effective. One interviewee said, “I think a lot of Hispanics have a lot of trust when they do something in a group. So, if there could be a clinic group or something at WIC [Women, Infants and Children social services center], that had an info session and kits, I think a lot of people would sign up and they would be supported. There could be a support group that could discuss the process and results.” (SL, female) Other participants underscored the importance of leveraging word-of-mouth and multi-generational channels of communication. For example, one participant said, “knowledge is power…The Latino community thrives with word-of-mouth, activities with family; a multi-generational approach” (EL, female).

### Recommendations for improving study procedures

Participants made several recommendations to support Hispanic participants. Common suggestions were to streamline the consent and results-reporting process (12%), to create a video explaining the screening process and the role of genetics in health, preferably from a provider’s perspective (10%), and to create a video that demonstrates how to complete the sample kit and how it is processed (14%). For the survey, 12% reported wanting a statement about whether and how not knowing family history might impact their results. Other participants desired a system to track the location of the mailed sample; one participant noted, “Maybe having an app where it tracks the process because it was a really long time and you just thought they lost it…checkpoints of where you are in the process like, HOP got your information, your information is being tested…or a text, like when you receive a text from [online vendors]….” (EL, female) Other participants wanted to have more upfront information about what supports, resources, and next steps would be available if they received abnormal results. Some focus group participants suggested that an incentive, in the form of a gift card or raffle, could help overcome barriers to participation (no monetary incentives are currently offered).

## Discussion

In seven focus groups, Hispanic participants in the HOP genetic screening cohort reported having a family member who was impacted by cancer, receiving no-cost genetic screening, and the trustworthy reputation of OHSU as strong motivators to enroll in HOP. Identified barriers to participating included lack of understanding or familiarity with research; immigration status concerns; and navigating technology. Following enrollment, participants reported difficulty filling out family history questions on the surveys as a barrier to completing participation. To improve Hispanic recruitment and enrollment, focus group participants recommended that study recruitment occur at community events, retail outlets, clinics, or schools using multimodal approaches (e.g., in-person and print materials). Participants also recommended a simplified consenting process, videos to demonstrate study tasks and the importance of genetics in disease risk, real-time sample tracking, and monetary participant incentives. The HOP study team will use these findings to refine its approach to recruitment. Findings may also be useful to inform other efforts to bolster Hispanic participation in clinical trials and research. As the Hispanic population is projected to account for about 25% of the US population by the year 2060, understanding how to effectively recruit Hispanic individuals in biomedical research will become increasingly important [10].

Participants in the focus groups expressed a preference for community-oriented recruitment methods. They highlighted the effectiveness of multimodal outreach at community events and engagement through established and trusted entities like clinics or community organizations. Unlike mass media recruitment, which includes methods like social media advertising primarily used by HOP, community-based recruitment involves reaching out to potential participants through sources they trust, such as their healthcare provider or familiar community organization. This aligns with prior research indicating that community-based strategies, such as involving community partners, employing bilingual and culturally sensitive research staff, fostering continuous engagement and participant-staff relationships, and embracing Hispanic cultural values, enhance inclusivity and help build trust [6,11].

Participants also recommended providing additional incentives for participation beyond offering no-cost genetic screening and genetic counseling and support for participants with positive results. Previous research has demonstrated that incentives can boost study participation. A recent systematic review that included six randomized clinical trials demonstrated that monetary incentives offered to participants boosted response rates by 27% (RR: 1.27; 95% CI: 1.04, 1.55; *P* = 0.02) and consent rates by 44% (RR: 1.44; 95% CI: 1.11, 1.85; *P* = 0.006) [12]. Walter and colleagues found that in hypothetical scenarios, requested payments differed significantly by racial and ethnic group, with Hispanic respondents requesting more payment than non-Hispanic White respondents [13]. This likely reflects the relatively high burden of participation for some Hispanic respondents.

The NASEM report concluded that many of the barriers to participation in clinical trials and research can be surmounted by actions taken by research teams, funders, and policymakers [2]. The HOP study team plans to implement changes to its study processes, including recruitment, in response to the insights from the focus groups. First, the HOP team plans to create videos that describe the importance of genetics in disease risk and cancer prevention and that demonstrate how to collect a saliva sample; these videos will incorporate responses to focus group-identified concerns and hesitations. Moreover, a new smartphone-based application that enables more interaction related to sample tracking will be offered to participants who have provided a saliva sample. Finally, the HOP research team plans to partner with key Hispanic-serving clinics and organizations to facilitate recruitment. HOP focus group participants specifically identified clinics as potential recruitment channels, which could enable targeted, broad-scale recruitment, using available clinic records. These partnerships can leverage name-recognition and trusted relationships and can provide a low-cost opportunity to reach large numbers of prospective Hispanic participants and provide direct assistance on how to download and interact with the HOP app.

This study had several strengths. Of the seven focus groups we conducted, two were held in Spanish, and five were held in English. This allowed us to understand the breadth of barriers and facilitators to study participation among both English- and Spanish-speaking Hispanic participants. Given that 38% of Hispanic adults in the United States mainly use Spanish, the perspectives of Spanish-preferring adults are critical to understanding Hispanic perspectives as a whole [14]. Because the study had already recruited over 40,000 participants, we were able to identify facilitators from those who had participated. Finally, the ongoing HOP study recruitment will allow our study team to implement and pilot-test recommended strategies.

Our study also had some limitations. First, consistent with our positive deviance approach, we intentionally limited our focus group recruitment to individuals who had participated in HOP; however, this inherently omitted the perspective of individuals who had not participated, which means that we may not have accurately captured barriers that truly prevented participation. Nevertheless, our participants identified several barriers and approaches that they perceived could bolster the recruitment of Hispanic participants. Moreover, our recruitment of participants identified challenges unique to study participation (e.g., experience with the app) that would likely not have been revealed by non-participants. Our focus group questions were specific to the HOP study, which included an at-home collection of a saliva sample and a self-administered questionnaire, and to Hispanic participant recruitment; thus, our findings may not be generalizable to studies with other procedures or requirements or to other racial and ethnic subgroups. Finally, our sample included a few male participants, whose perspectives may differ from those of females.

## Conclusion

Through focus groups, Hispanic participants of an Oregon-based cancer and cardiovascular disease genetics study identified key barriers and motivators to study participation. Participants recommended that study recruitment occur at community events, clinics, and schools using a simplified consenting process, videos to demonstrate study tasks, real-time sample tracking, and monetary incentives. Our findings can inform efforts to achieve appropriate representation of Hispanic populations in clinical trials and research, enable the findings of this research to benefit all populations, and thereby deliver on the promise of the research enterprise.

## Supporting information

Coronado et al. supplementary material Coronado et al. supplementary material

## Supplementary material

For supplementary material accompanying this paper visit https://doi.org/10.1017/S2059866124005442. click here to view supplementary material
