# Supplementary material for: Improving representation of Hispanic adults in a population-based cancer genetics cohort: Qualitative findings from the Healthy Oregon Project
Source: J Clin Transl Sci. 2024 May 22;8(1):e99. doi: 10.1017/cts.2024.544 (PMC11626583; doi:10.1017/cts.2024.544)
Supplement: Coronado et al. supplementary material [file S2059866124005442sup001.docx]

**Supplementary Table 1.** Study Question Guide

| **Domain** | **Questions** | **Probes** |
| --- | --- | --- |
| Decision-making Process | How did you find out about the Healthy Oregon Project (HOP)? | Did you join the very first time you heard about HOP or how many times/sources did you hear about it from?  Can you recall what the advertisement said? |
|  | What made you sign-up? | What was the most important reason for your decision?  What motivated you to sign up and go through the entire process?  Who did you talk to about joining HOP? |
|  | Thinking of how you heard about HOP, is there another way you would have preferred to hear about HOP? | How would you want to receive education about genetic screening? |
| Information Quality | For those of you who saw an ad or post on social media, what did you think about the post/ad? | What part of the message was most impactful or influenced you to join? |
|  | When you saw the post/ad did you have any unanswered questions or felt like important information was missing? | If so, what questions/information did you want to see?  Did you get that information before you joined (if so, how) (if not, what made you join anyways)? |
| Understanding of Information | What was your overall experience during the sign-up (including app download) process? | Was there anything you were confused or unclear about?  What did you think about how long it took you to go through the process? |
|  | Did someone help you go through the sign-up process? |  |
|  | Overall, how well would you say you understood the study when you decided to join? |  |
| Emotional Response and Trust | Now, I’d like you to think back to how you felt right after hearing about the study. What were you feeling? | Did you feel anxious? Did you hesitate? If so, what were you anxious, hesitant about?  How did you deal with your feelings (look up information, talk to someone, etc.)? |
|  | Who do you trust to provide you with the information you need about genetic screening? |  |
| General Experience and Acceptability | The first step in the HOP study was to answer questions about your family history of cancer. What made you interested in starting the family history questions for the HOP study? | Do you remember how interested you were in genetic screening before answering the questions? |
|  | The second step in the HOP study was to request your HOP kit for your saliva sample. Did you have any questions or concerns about mailing your sample? What did you do when you received the kit in the mail? | Did you open it right away? Set it aside? Any new concerns arise? |
| Respect | Could you tell me about anything that made you feel respected when you were making that decision to join? | Think about interactions with researchers, research study materials, how easy/hard it was to get the information you needed.  What was it about that that made you feel respected? |
| Family | Have other family members or friends also joined HOP? If yes, how did that influence your decision? |  |
|  | Have you talked with anyone in your family about genetic screening and what you have learned? | How did you tell them (phone/face to face/letter/etc.,)?  How did the conversation go?  How did they respond to the information and your decision? |
|  | Have any of the family members you’ve told been screened? Or any planning to get screened? | Through the HOP study, or elsewhere? Why/why not?  Did you tell your family members that they could get free genetic screening through the study? If so, what was their reaction? |
|  | If you were going to give advice to someone in your family considering genetic screening, how would you help them think about the possible benefits and challenges? |  |
| General Improvements | Thinking about the whole process of learning about HOP and signing up, is there anything we can improve? |  |
|  | If you were to design a way to get more people who are Hispanic/Latino to join HOP, how would you do it differently? |  |
| Closing | Is there anything else you would like to share with me about your experience with HOP? |  |

*********

**Supplementary Table 2.** Differences in response by language of focus group (English vs. Spanish)

| **Domain** | **Difference in English- and Spanish-language focus groups** |
| --- | --- |
| **General Motivators for participation** | |
| **Trust in Oregon Health and Science University** | Both Spanish and English-speaking participants expressed trust in OHSU and recognized its positive impact on the community. However, English-speaking participants reported higher levels of trust in OHSU. |
| **Family History of Cancer** | Both groups shared similar experiences and concerns regarding personal or familial encounters with cancer, indicating a universal recognition of its significance. |
| **Family/Friends Influence** | Participants from both language groups highlighted the importance of support from family and friends in their decision-making process, suggesting a shared value placed on social networks. |
| **Research** | English-speaking participants seemed more actively engaged with research, as evidenced by their higher participation rates and inclination towards reading research articles. Conversely, Spanish-speaking participants expressed a need for increased representation of Latino/a individuals in research, suggesting a perception of underrepresentation and limited understanding of the world of research and its processes. |
| **Prevention** | English-speaking participants appeared more concerned about the lack of detailed family history and its implications for cancer prevention. This suggests a slightly higher level of uncertainty and fear surrounding cancer risk factors compared to their Spanish-speaking counterparts. |
| **Cost** | While both groups appreciated the availability of free testing kits, there were slight variations in emphasis. Spanish-speaking participants placed slightly more importance on cost, considering the higher proportion of responses indicating free kits as a motivating factor. |
| **General Barriers** | |
| **Technology literacy, immigration status, mistrust in research** | Both Spanish and English-speaking participants shared similar concerns regarding technology literacy, immigration status, and mistrust in research experienced by the Hispanic community. |
| **Fear** | Spanish-speaking participants appeared to express more fears associated with participating in the study, noting fear of the results and lack of knowledge around cancer and treatment. |
| **Literacy-related barriers** | Spanish-speaking participants showed a slightly higher emphasis on literacy-related barriers, including understanding study information and consent forms. |
| **Recruitment** | |
| **Events at trusted locations** | Both Spanish and English-speaking participants agreed on the effectiveness of trusted location advertising, provider recommendations, community events, and partnerships in improving recruitment efforts.  Promoting sign-ups and kit completion at Latino community events, fairs, and markets was strongly endorsed by both Spanish and English-speaking participants. |
| **Emphasis in recruitment** | English-speaking participants placed slightly more emphasis on education and trust-building efforts, while Spanish-speaking participants emphasized the importance of emphasizing the no-cost aspect of participation in advertisements. |
| **Role of community organizations** | The idea of using school district emails, wellness centers, and sending brochures home with kids received notable endorsement, with 9 English-speaking participants supporting it, while only 1 Spanish-speaking participant endorsed it. |
| **Study Improvements** | |
| **Clearer process** | English-speaking participants expressed a desire for clearer flow/order of survey completion and emphasized the need for a disclaimer regarding the impact of not knowing family history on results. |
| **Streamlined consent** | Both groups suggested streamlining consent and result processes to make them more user-friendly. |
| **Survey modalities** | English-speaking participants noted that surveys would be easier to complete on laptops than phones and suggested offering multiple ways to complete the surveys. |
| **Alternate sign-up methods** | Spanish-speaking participants suggested offering alternative sign-up methods aside from downloading the application on a phone. |
| **Video content** | Spanish-speaking participants favored videos showing how to complete the kit and explaining the registration process, while English-speaking participants preferred videos explaining genetics and the testing process from a provider perspective. |
| **Follow-up resources** | Spanish-speaking participants emphasized the importance of follow-up resources and next steps for abnormal results, as well as including guidelines for other screenings when receiving results.  English-speaking participants also mentioned the importance of kit tracking and follow-up resources, though to a slightly lesser extent. |
